# A Funny Resolution

**Published:** 1866-12

**Authors:** 


### A FUNNY RESOLUTION.

IN the proceedings of the "Connecticut Valley Dental Association," published in this number of the "REGISTER," is the following:

"*Resolved*, That this Association do not consider the nitrous oxyd made by Sprague's arrangement any purer than may be produced by the apparatus not interfering with his patent, which applies only to the convenience of the operator in regulating the flame."

Now, with all due deference and respect,—since the Irishmen resolved to have a new jail, to build it out of the materials of the old jail, and to keep the prisoners in the old, till the new one was finished—such a platitude as this resolution has not been passed by a deliberative body.

We are not the champion of Mr. Sprague. The quality and price of his apparatus indicate that he can take care of himself. But the *people* have a right to protection against the perversion of science. Nor have we serious objection to the main statement of the resolution, if it means just what it says. The Association does not consider the nitrous oxyd made by Sprague's apparatus any purer than MAY BE produced by apparatus without a heat regulator. A porcelain glue-pot, a discarded pickle-jar, an abandoned nail-keg, and a relinquished fruit-can can be so arranged that pure nitrous oxyd *may be* obtained now and then, by their aid; but it is just barely possible, and that is all that the resolution claims for the apparatus it refers to.

An engineer *may* run a steamer across the Atlantic without a steam gauge; but only fools would, knowingly, cross a river on a ferry-boat so arranged. As well might it be said that the steam gauge "applies only to the convenience of the engineer," in regulating the fire, as claimed that Sprague's regulator "applies only to the convenience of the operator." A steam gauge *is* a "convenience" to the engineer. He can ascertain the pressure of the steam with more ease, and *perhaps more accurately*, than by looking at the fire. But the gauge is also a "*convenience*" to the passengers, unless they are ready to take the risk of a heavenward flight on a cloud of steam. But the passengers are quite as safe on a steamer without a gauge, as a patient is inhaling gaseous matter generated by the decomposition of nitrate of ammonia without a heat regulator. For the explanation of the danger and its sources, see an article in the present number entitled, "Nitrous Oxyd as an Anæsthetic."

A case in point has just been told us by the parties concerned: Two of our Dental friends, well posted in the science of our profession, (the Connecticut Valley Dental Association would regard them as the equals of their best members) conluded to experiment with the gas. They bought their apparatus at a standard dental depot. It had no regulator. One of them administered the gas to the other, with alarming, and as they believe, with nearly fatal results. The heat had run too high, at least a part of the time, while generating the gas, and *nitric*, instead of *nitrous* oxyd was formed. We know of a number who are suffering from laryngeal, and bronchial irritation, from the same cause.

Suppose physicians and surgeons had to prepare their own chloroform as they use it. Does any sane man suppose the results would be safe or satisfactory? Yet there is less liability to mistake in making chloroform than in making nitrous oxyd. We never yet met with a case in which we were afraid to give chloroform that the patient could not find a medical or dental student, of but a few months' study, willing and anxious to administer it. This is in accordance with human nature. Only new recruits are fool-hardy; *veterans* are *brave*, but never reckless.

Several years ago, a merry group of us were on the "Maid of the Mist." Cheerful songs and laughter rung out as from the mouths of released school children. Our attention was arrested by the attitude of the Captain. He stood alone, with compressed lips—eyes and nostrils dilated—his whole demeanor giving evidence of agonizing attention. We were making a single trip, and realized only a pleasure ride on a steamboat. He was going thus, hour by hour, for months, and *knew he was playing with Niagara.* So it is with anæsthetics. Those who know the least have the fewest fears. "Fools rush in where angels fear to tread," is not the correct quotation, but it expresses the idea.

But does so short a resolution need such a long notice? It was passed at a meeting of a large, active, *good* association of our profession. If so many, in one locality are in error, there is a call for light; and there is no danger from too much. We are told that the resolution passed unanimously. We have read of a flock of sheep that *unanimously* drowned themselves in the sea, when their blind bell-weather jumped over the cliffs; and we have always believed that, if they had possessed a clear idea of what they were doing, there would have been some dissenters among them. So we believe, if there had been *one* good practical chemist in the Connecticut Valley Association, the resolution, if passed at all, would have had a different vote. W.

